# [(1*R*,4*S*)-(+)-3-Benzoyl-1,7,7-trimethyl­bicyclo­[2.2.1]heptan-2-olato-κ^2^ *O* ^2^,*O* ^3^](η^4^-norbornadiene)rhodium(I)

**DOI:** 10.1107/S160053681002667X

**Published:** 2010-07-10

**Authors:** Mohamed Anoir Harrad, Brahim Boualy, Mustapha Ait Ali, Larbi El Firdoussi, Corrado Rizzoli

**Affiliations:** a Laboratoire de Chimie de Coordination, Faculté des Sciences-Semlalia, BP 2390, 40001 Marrakech, Morocco; b Dipartimento di Chimica Generale ed Inorganica, Chimica Analitica, Chimica Fisica, Universitá degli Studi di Parma, Viale G. P. Usberti 17/A, I-43100 Parma, Italy

## Abstract

In the title complex mol­ecule, [Rh(C_17_H_19_O_2_)(C_7_H_8_)], the rhodium(I) metal centre is coordinated by the O atoms of a benzoyl­camphorate anion and the C=C bonds of the norbornadiene mol­ecule into a slightly distorted square-planar coordination geometry. The six-membered chelate ring is essentially planar (r.m.s. deviation = 0.0378 Å) and forms a dihedral angle of 31.67 (11)° with the phenyl ring.

## Related literature

For the synthesis and properties of rhodium complexes in enanti­oselective transformations, see: Noyori (1994 ▶); Breuzard *et al.* (2000 ▶); Bernard *et al.* (2001 ▶). For the chemistry and applications of camphor-derived compounds, see: Togni (1990 ▶); Togni *et al.* (1993 ▶); Guo & Sadler (1999 ▶). For the synthesis, structure and applications of transition metal complexes in catalytic asymmetric reactions, see: Naili *et al.* (2000 ▶); Ait Ali, Allaoud *et al.* (2000 ▶); Fdil *et al.* (2002 ▶). For related structures, see: Spannenberg *et al.* (2002 ▶); Ait Ali, El Firdoussi *et al.* (2000 ▶); Ait Ali *et al.* (2001 ▶, 2006 ▶); El Firdoussi *et al.* (2007 ▶). For a description of the Cambridge Structural Database, see: Allen (2002 ▶).
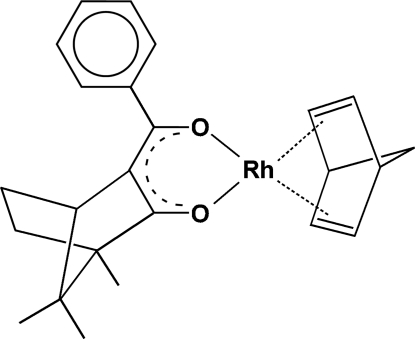


## Experimental

### 

#### Crystal data

[Rh(C_17_H_19_O_2_)(C_7_H_8_)]
*M* *_r_* = 450.37
Orthorhombic, 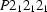

*a* = 6.4755 (11) Å
*b* = 8.2817 (13) Å
*c* = 38.320 (6) Å
*V* = 2055.0 (6) Å^3^
*Z* = 4
Mo *K*α radiation
μ = 0.85 mm^−1^
*T* = 295 K
0.33 × 0.16 × 0.10 mm

#### Data collection

Bruker SMART 1000 CCD diffractometer
Absorption correction: multi-scan (*SADABS*; Bruker, 1998 ▶) *T*_min_ = 0.855, *T*_max_ = 0.937
21718 measured reflections
3990 independent reflections
3953 reflections with *I* > 2σ(*I*)
*R*_int_ = 0.040

#### Refinement

*R*[*F* ^2^ > 2σ(*F* ^2^)] = 0.034
*wR*(*F* ^2^) = 0.074
*S* = 1.27
3990 reflections
244 parameters
H-atom parameters constrained
Δρ_max_ = 0.59 e Å^−3^
Δρ_min_ = −1.11 e Å^−3^
Absolute structure: Flack (1983 ▶), 1643 Friedel pairs
Flack parameter: 0.03 (4)

### 

Data collection: *SMART* (Bruker, 1998 ▶); cell refinement: *SAINT-Plus* (Bruker, 1998 ▶); data reduction: *SAINT-Plus*; program(s) used to solve structure: *SIR97* (Altomare *et al.*, 1999 ▶); program(s) used to refine structure: *SHELXL97* (Sheldrick, 2008 ▶); molecular graphics: *ORTEP-3 for Windows* (Farrugia, 1997 ▶) and *SCHAKAL97* (Keller, 1997 ▶); software used to prepare material for publication: *SHELXL97* and *PARST95* (Nardelli, 1995 ▶).

## Supplementary Material

Crystal structure: contains datablocks global, I. DOI: 10.1107/S160053681002667X/gk2290sup1.cif

Structure factors: contains datablocks I. DOI: 10.1107/S160053681002667X/gk2290Isup2.hkl

Additional supplementary materials:  crystallographic information; 3D view; checkCIF report

## Figures and Tables

**Fig. 1.**
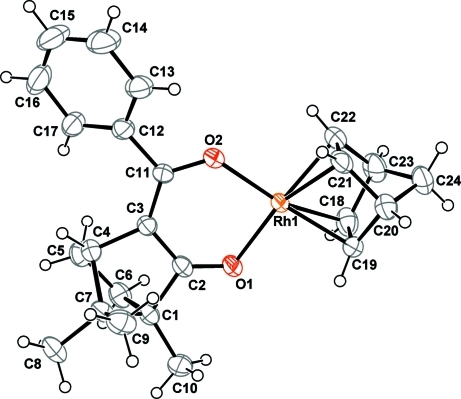
The molecular structure of the title compound. Displacement ellipsoids are drawn at the 40% probability level.

**Fig. 2.**
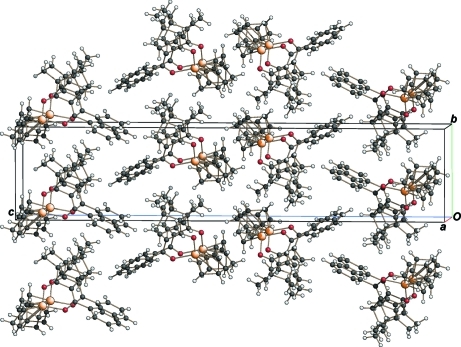
Crystal packing of the title compound viewed approximately along the a axis.

## supplementary crystallographic information

### Comment

Rhodium complexes are widely used in organic chemistry due to their ability to mediate numerous transformations of organic molecules, often in catalytic mode. In particular, rhodium complexes of chiral ligands have shown to perform highly enantioselective transformations (Noyori, 1994; Breuzard *et al.*, 2000; Bernard *et al.*, 2001). Camphor-derived 1,3-diketonato ligands are a potentially attractive class of ligands in organometallic development, because these compounds are readily synthesized and easily varied (Togni, 1990; Togni *et al.*, 1993). Moreover, some of their transition metal complexes can be used as therapeutic drugs (Guo & Sadler, 1999). As a contribution to our research programs aimed at the preparation of transition metal complexes (Spannenberg *et al.*, 2002; Ait Ali, El Firdoussi *et al.*, 2000; Ait Ali *et al.*, 2001, 2006; El Firdoussi *et al.*, 2007) and their application in catalytic asymmetric reactions (Naili *et al.*, 2000; Ait Ali, Allaoud *et al.*, 2000; Fdil *et al.*, 2002), we report here the synthesis and crystal structure of the title compound.

In the mononuclear title complex molecule (Fig. 1), the rhodium(I) metal atom assumes a slightly tetrahedrally distorted square-planar coordination geometry provided by the O atoms of the chelating benzoylcamphorato anion and the centroids of the C═C double bonds of the norbornadiene molecule (maximum displacement 0.078 (5) Å for the centroid of the C18═C19 bonds). The RhO_2_C_3_ six-membered chelate ring is essentially planar (*r.m.s.* deviation = 0.0378 Å) and forms a dihedral angle of 31.67 (11)° with the C12–C17 phenyl ring. The C–O (O1–C2 = 1.265 (5) Å; O2–C11 = 1.298 (5) Å) and C–C (C2–C3 = 1.427 (6) Å; C3–C11 = 1.402 (5) Å) bond lengths pattern within the metallacycle indicates a high degree of π-delocalization. The Rh–O bond lengths (2.047 (3) and 2.059 (2) Å) are not significantly different from those observed in the closely related compound (cycloocta-1,5-diene)[(1*R*)-(+)-3-benzoyl-camphoryl]rhodium(I) (2.047 (3) and 2.059 (2) Å; Spannenberg *et al.*, 2002). The Rh–C (mean value 2.113 (4) Å) and the donor C═C double bonds distances (mean value 1.388 (7) Å) involving the norbornadiene molecule are in agreement with the range of values observed in 286 related structures (mean values: Rh–C, 2.164 Å; C═C, 1.384 Å; Cambridge Structural Database; Version 5.31, November 2009; Allen, 2002). The crystal packing (Fig. 2) is stabilized only by van der Waals interactions.

### Experimental

A solution of [Rh(norbornadiene)Cl]_2_ (0.18 mmol, 100 mg) in THF (10 ml) was added to a suspension of (1*R*)-(+)-3-benzoylcamphor (0.32 mmol, 83.4 mg) and Na_2_CO_3_ (0.94 mmol, 100 mg) in THF (10 ml). The mixture was stirred for 3 h at room temperature, then it was evaporated to dryness under reduced pressure. The residue was extracted with CH_2_Cl_2_ (3 × 10 ml), and the recovered filtrate was evaporated to dryness to give an orange solid (yield 86%). Crystals suitable for X-ray analysis were obtained by slow evaporation of a diethyl ether solution.

### Refinement

All H atoms were placed at calculated positions and refined using the riding model approximation, with C—H = 0.93–0.97 Å, and with *U*_iso_(H) = 1.2 *U*_eq_(C) or 1.5 *U*_eq_(C) for methyl H atoms. The absolute configuration was assigned on the basis of the known absolute configuration of the starting material and confirmed by anomalous scattering effects.

### Crystal data

**Table:**

[Rh(C_17_H_19_O_2_)(C_7_H_8_)]	*F*(000) = 928
*M**_r_* = 450.37	*D*_x_ = 1.456 Mg m^−^^3^
Orthorhombic, *P*2_1_2_1_2_1_	Mo *K*α radiation, λ = 0.71073 Å
Hall symbol: P 2ac 2ab	Cell parameters from 772 reflections
*a* = 6.4755 (11) Å	θ = 5.2–24.8°
*b* = 8.2817 (13) Å	µ = 0.85 mm^−^^1^
*c* = 38.320 (6) Å	*T* = 295 K
*V* = 2055.0 (6) Å^3^	Block, orange
*Z* = 4	0.33 × 0.16 × 0.10 mm

### Data collection

**Table:**

Bruker SMART 1000 CCD diffractometer	3990 independent reflections
Radiation source: fine-focus sealed tube	3953 reflections with *I* > 2σ(*I*)
graphite	*R*_int_ = 0.040
ω scans	θ_max_ = 26.0°, θ_min_ = 1.1°
Absorption correction: multi-scan (*SADABS*; Bruker, 1998)	*h* = −7→7
*T*_min_ = 0.855, *T*_max_ = 0.937	*k* = −10→10
21718 measured reflections	*l* = −47→47

### Refinement

**Table:**

Refinement on *F*^2^	Secondary atom site location: difference Fourier map
Least-squares matrix: full	Hydrogen site location: inferred from neighbouring sites
*R*[*F*^2^ > 2σ(*F*^2^)] = 0.034	H-atom parameters constrained
*wR*(*F*^2^) = 0.074	*w* = 1/[σ^2^(*F*_o_^2^) + (0.017*P*)^2^ + 2.5414*P*] where *P* = (*F*_o_^2^ + 2*F*_c_^2^)/3
*S* = 1.27	(Δ/σ)_max_ = 0.001
3990 reflections	Δρ_max_ = 0.59 e Å^−^^3^
244 parameters	Δρ_min_ = −1.11 e Å^−^^3^
0 restraints	Absolute structure: Flack (1983), 1643 Friedel pairs
Primary atom site location: structure-invariant direct methods	Flack parameter: 0.03 (4)

### Special details

**Table:**

Refinement. Refinement of *F*^2^ against ALL reflections. The weighted *R*-factor *wR* and goodness of fit *S* are based on *F*^2^, conventional *R*-factors *R* are based on *F*, with *F* set to zero for negative *F*^2^. The threshold expression of *F*^2^ > σ(*F*^2^) is used only for calculating *R*-factors(gt) *etc*. and is not relevant to the choice of reflections for refinement. *R*-factors based on *F*^2^ are statistically about twice as large as those based on *F*, and *R*- factors based on ALL data will be even larger.

### Fractional atomic coordinates and isotropic or equivalent isotropic displacement parameters (Å^2^)

**Table:**

	*x*	*y*	*z*	*U*_iso_*/*U*_eq_	
Rh1	0.56683 (5)	0.36256 (3)	0.081995 (8)	0.03291 (9)	
O1	0.3616 (4)	0.1728 (3)	0.08213 (8)	0.0400 (6)	
O2	0.5107 (4)	0.4086 (3)	0.13360 (7)	0.0398 (7)	
C1	0.1414 (6)	−0.0255 (5)	0.11061 (11)	0.0368 (9)	
C2	0.2775 (6)	0.1238 (5)	0.11000 (10)	0.0344 (8)	
C3	0.2779 (6)	0.1910 (5)	0.14431 (10)	0.0318 (8)	
C4	0.1389 (6)	0.0796 (5)	0.16545 (11)	0.0355 (9)	
H4	0.1590	0.0847	0.1908	0.043*	
C5	−0.0856 (7)	0.1121 (6)	0.15294 (11)	0.0471 (10)	
H5A	−0.1853	0.0554	0.1673	0.056*	
H5B	−0.1171	0.2266	0.1532	0.056*	
C6	−0.0825 (8)	0.0446 (6)	0.11516 (12)	0.0460 (10)	
H6A	−0.1088	0.1296	0.0983	0.055*	
H6B	−0.1855	−0.0394	0.1123	0.055*	
C7	0.1856 (7)	−0.0886 (5)	0.14854 (12)	0.0380 (9)	
C8	0.0410 (9)	−0.2252 (5)	0.16093 (13)	0.0531 (12)	
H8A	−0.1000	−0.1941	0.1571	0.080*	
H8B	0.0628	−0.2445	0.1854	0.080*	
H8C	0.0703	−0.3219	0.1480	0.080*	
C9	0.4082 (7)	−0.1450 (6)	0.15381 (13)	0.0512 (10)	
H9A	0.5013	−0.0621	0.1461	0.077*	
H9B	0.4317	−0.2415	0.1405	0.077*	
H9C	0.4315	−0.1667	0.1781	0.077*	
C10	0.1716 (7)	−0.1380 (6)	0.07994 (14)	0.0571 (12)	
H10A	0.0806	−0.2289	0.0822	0.086*	
H10B	0.3121	−0.1749	0.0795	0.086*	
H10C	0.1413	−0.0815	0.0587	0.086*	
C11	0.3863 (6)	0.3304 (4)	0.15408 (10)	0.0327 (9)	
C12	0.3729 (7)	0.4015 (5)	0.19004 (11)	0.0394 (10)	
C13	0.5477 (10)	0.4824 (5)	0.20309 (12)	0.0527 (12)	
H13	0.6684	0.4859	0.1900	0.063*	
C14	0.5404 (13)	0.5571 (7)	0.23555 (14)	0.0759 (19)	
H14	0.6574	0.6083	0.2442	0.091*	
C15	0.3625 (13)	0.5558 (8)	0.25484 (16)	0.086 (2)	
H15	0.3576	0.6085	0.2763	0.103*	
C16	0.1911 (12)	0.4765 (7)	0.24245 (15)	0.0758 (19)	
H16	0.0718	0.4738	0.2559	0.091*	
C17	0.1928 (9)	0.3994 (6)	0.20983 (12)	0.0535 (12)	
H17	0.0751	0.3476	0.2016	0.064*	
C18	0.5765 (9)	0.4075 (6)	0.02782 (11)	0.0517 (11)	
H18	0.4397	0.3876	0.0218	0.062*	
C19	0.7252 (7)	0.2940 (5)	0.03643 (11)	0.0413 (10)	
H19	0.7076	0.1827	0.0375	0.050*	
C20	0.9252 (8)	0.3882 (5)	0.04392 (11)	0.0466 (10)	
H20	1.0552	0.3276	0.0436	0.056*	
C21	0.8629 (7)	0.4696 (5)	0.07839 (13)	0.0441 (10)	
H21	0.9148	0.4467	0.1005	0.053*	
C22	0.7137 (8)	0.5842 (5)	0.07018 (13)	0.0526 (13)	
H22	0.6459	0.6529	0.0856	0.063*	
C23	0.6835 (9)	0.5738 (6)	0.03026 (13)	0.0570 (13)	
H23	0.6157	0.6652	0.0187	0.068*	
C24	0.9039 (10)	0.5307 (6)	0.01829 (13)	0.0604 (14)	
H24A	1.0027	0.6163	0.0227	0.072*	
H24B	0.9101	0.4974	−0.0060	0.072*	

### Atomic displacement parameters (Å^2^)

**Table:**

	*U*^11^	*U*^22^	*U*^33^	*U*^12^	*U*^13^	*U*^23^
Rh1	0.03562 (15)	0.03097 (13)	0.03214 (14)	−0.00890 (14)	0.00359 (13)	−0.00053 (14)
O1	0.0433 (15)	0.0385 (15)	0.0383 (14)	−0.0161 (11)	0.0057 (13)	−0.0017 (14)
O2	0.0494 (18)	0.0328 (15)	0.0372 (15)	−0.0137 (12)	0.0057 (12)	−0.0036 (11)
C1	0.031 (2)	0.036 (2)	0.043 (2)	−0.0109 (17)	0.0029 (17)	−0.0011 (18)
C2	0.0289 (18)	0.031 (2)	0.043 (2)	−0.0044 (18)	0.0009 (15)	0.0034 (19)
C3	0.031 (2)	0.0294 (19)	0.035 (2)	−0.0016 (15)	0.0032 (16)	0.0002 (16)
C4	0.035 (2)	0.037 (2)	0.035 (2)	−0.0034 (16)	0.0016 (16)	0.0032 (17)
C5	0.034 (2)	0.050 (3)	0.057 (3)	−0.002 (2)	0.007 (2)	0.002 (2)
C6	0.033 (2)	0.052 (3)	0.053 (3)	−0.008 (2)	−0.002 (2)	0.008 (2)
C7	0.035 (2)	0.029 (2)	0.050 (2)	−0.0058 (17)	0.0006 (18)	0.0041 (17)
C8	0.055 (3)	0.041 (2)	0.064 (3)	−0.014 (2)	0.005 (3)	0.013 (2)
C9	0.044 (3)	0.040 (2)	0.069 (3)	0.004 (3)	−0.003 (2)	0.000 (2)
C10	0.059 (3)	0.052 (2)	0.060 (3)	−0.026 (2)	0.010 (2)	−0.019 (3)
C11	0.039 (2)	0.0233 (19)	0.036 (2)	0.0011 (15)	0.0036 (16)	0.0002 (15)
C12	0.057 (3)	0.027 (2)	0.035 (2)	0.0042 (17)	0.0007 (18)	0.0028 (16)
C13	0.076 (4)	0.041 (2)	0.041 (2)	−0.006 (3)	−0.006 (3)	−0.0033 (18)
C14	0.117 (6)	0.061 (3)	0.049 (3)	−0.015 (4)	−0.019 (4)	−0.013 (3)
C15	0.149 (7)	0.062 (4)	0.047 (3)	−0.001 (4)	0.009 (4)	−0.025 (3)
C16	0.122 (6)	0.060 (3)	0.046 (3)	0.019 (4)	0.031 (3)	−0.001 (3)
C17	0.075 (3)	0.044 (3)	0.042 (2)	0.006 (2)	0.014 (2)	−0.002 (2)
C18	0.047 (2)	0.072 (3)	0.037 (2)	−0.014 (3)	−0.003 (2)	0.007 (2)
C19	0.053 (3)	0.039 (2)	0.031 (2)	−0.011 (2)	0.0085 (19)	−0.0046 (18)
C20	0.040 (2)	0.048 (2)	0.052 (2)	−0.006 (2)	0.008 (2)	0.0018 (19)
C21	0.046 (2)	0.045 (2)	0.040 (2)	−0.0219 (18)	0.000 (2)	0.000 (2)
C22	0.064 (3)	0.033 (2)	0.061 (3)	−0.013 (2)	0.021 (2)	0.002 (2)
C23	0.068 (3)	0.049 (3)	0.054 (3)	0.001 (3)	0.005 (3)	0.025 (2)
C24	0.070 (4)	0.061 (3)	0.050 (3)	−0.020 (3)	0.015 (3)	0.009 (2)

### Geometric parameters (Å, °)

**Table:**

Rh1—O2	2.047 (3)	C10—H10A	0.9600
Rh1—O1	2.059 (2)	C10—H10B	0.9600
Rh1—C19	2.103 (4)	C10—H10C	0.9600
Rh1—C18	2.110 (4)	C11—C12	1.501 (5)
Rh1—C22	2.116 (4)	C12—C17	1.391 (7)
Rh1—C21	2.116 (4)	C12—C13	1.407 (7)
O1—C2	1.265 (5)	C13—C14	1.390 (7)
O2—C11	1.298 (5)	C13—H13	0.9300
C1—C10	1.512 (6)	C14—C15	1.369 (10)
C1—C2	1.519 (5)	C14—H14	0.9300
C1—C7	1.571 (6)	C15—C16	1.374 (10)
C1—C6	1.572 (6)	C15—H15	0.9300
C2—C3	1.427 (6)	C16—C17	1.403 (7)
C3—C11	1.402 (5)	C16—H16	0.9300
C3—C4	1.522 (5)	C17—H17	0.9300
C4—C5	1.554 (6)	C18—C19	1.386 (7)
C4—C7	1.566 (6)	C18—C23	1.545 (7)
C4—H4	0.9800	C18—H18	0.9300
C5—C6	1.552 (6)	C19—C20	1.539 (6)
C5—H5A	0.9700	C19—H19	0.9300
C5—H5B	0.9700	C20—C21	1.537 (6)
C6—H6A	0.9700	C20—C24	1.541 (6)
C6—H6B	0.9700	C20—H20	0.9800
C7—C9	1.529 (6)	C21—C22	1.390 (7)
C7—C8	1.543 (6)	C21—H21	0.9300
C8—H8A	0.9600	C22—C23	1.544 (7)
C8—H8B	0.9600	C22—H22	0.9300
C8—H8C	0.9600	C23—C24	1.541 (8)
C9—H9A	0.9600	C23—H23	0.9800
C9—H9B	0.9600	C24—H24A	0.9700
C9—H9C	0.9600	C24—H24B	0.9700
			
O2—Rh1—O1	91.44 (11)	H10A—C10—H10B	109.5
O2—Rh1—C19	159.91 (16)	C1—C10—H10C	109.5
O1—Rh1—C19	96.36 (14)	H10A—C10—H10C	109.5
O2—Rh1—C18	157.38 (16)	H10B—C10—H10C	109.5
O1—Rh1—C18	98.99 (17)	O2—C11—C3	124.1 (4)
C19—Rh1—C18	38.40 (19)	O2—C11—C12	113.3 (3)
O2—Rh1—C22	97.18 (16)	C3—C11—C12	122.6 (3)
O1—Rh1—C22	162.36 (18)	C17—C12—C13	119.1 (4)
C19—Rh1—C22	80.64 (18)	C17—C12—C11	123.0 (4)
C18—Rh1—C22	67.9 (2)	C13—C12—C11	117.8 (4)
O2—Rh1—C21	98.38 (15)	C14—C13—C12	120.2 (6)
O1—Rh1—C21	154.79 (15)	C14—C13—H13	119.9
C19—Rh1—C21	67.50 (18)	C12—C13—H13	119.9
C18—Rh1—C21	80.5 (2)	C15—C14—C13	120.5 (6)
C22—Rh1—C21	38.35 (19)	C15—C14—H14	119.7
C2—O1—Rh1	121.6 (3)	C13—C14—H14	119.7
C11—O2—Rh1	127.0 (2)	C14—C15—C16	119.8 (5)
C10—C1—C2	114.5 (3)	C14—C15—H15	120.1
C10—C1—C7	119.4 (4)	C16—C15—H15	120.1
C2—C1—C7	100.3 (3)	C15—C16—C17	121.3 (6)
C10—C1—C6	115.7 (4)	C15—C16—H16	119.4
C2—C1—C6	103.7 (3)	C17—C16—H16	119.4
C7—C1—C6	100.9 (3)	C12—C17—C16	119.1 (6)
O1—C2—C3	130.7 (4)	C12—C17—H17	120.5
O1—C2—C1	121.6 (4)	C16—C17—H17	120.5
C3—C2—C1	107.7 (3)	C19—C18—C23	106.2 (4)
C11—C3—C2	124.6 (4)	C19—C18—Rh1	70.5 (2)
C11—C3—C4	130.8 (4)	C23—C18—Rh1	96.4 (3)
C2—C3—C4	104.6 (3)	C19—C18—H18	126.9
C3—C4—C5	106.5 (3)	C23—C18—H18	126.9
C3—C4—C7	101.8 (3)	Rh1—C18—H18	100.7
C5—C4—C7	102.0 (3)	C18—C19—C20	106.6 (4)
C3—C4—H4	115.0	C18—C19—Rh1	71.1 (3)
C5—C4—H4	115.0	C20—C19—Rh1	96.8 (3)
C7—C4—H4	115.0	C18—C19—H19	126.7
C6—C5—C4	102.3 (4)	C20—C19—H19	126.7
C6—C5—H5A	111.3	Rh1—C19—H19	99.9
C4—C5—H5A	111.3	C21—C20—C19	99.3 (3)
C6—C5—H5B	111.3	C21—C20—C24	100.9 (4)
C4—C5—H5B	111.3	C19—C20—C24	101.2 (4)
H5A—C5—H5B	109.2	C21—C20—H20	117.5
C5—C6—C1	104.4 (4)	C19—C20—H20	117.5
C5—C6—H6A	110.9	C24—C20—H20	117.5
C1—C6—H6A	110.9	C22—C21—C20	106.7 (4)
C5—C6—H6B	110.9	C22—C21—Rh1	70.8 (2)
C1—C6—H6B	110.9	C20—C21—Rh1	96.3 (3)
H6A—C6—H6B	108.9	C22—C21—H21	126.7
C9—C7—C8	107.9 (4)	C20—C21—H21	126.7
C9—C7—C4	113.5 (4)	Rh1—C21—H21	100.5
C8—C7—C4	114.1 (4)	C21—C22—C23	105.9 (4)
C9—C7—C1	113.3 (4)	C21—C22—Rh1	70.8 (2)
C8—C7—C1	114.7 (4)	C23—C22—Rh1	96.1 (3)
C4—C7—C1	93.0 (3)	C21—C22—H22	127.0
C7—C8—H8A	109.5	C23—C22—H22	127.0
C7—C8—H8B	109.5	Rh1—C22—H22	100.6
H8A—C8—H8B	109.5	C24—C23—C22	101.0 (4)
C7—C8—H8C	109.5	C24—C23—C18	101.0 (4)
H8A—C8—H8C	109.5	C22—C23—C18	99.6 (3)
H8B—C8—H8C	109.5	C24—C23—H23	117.4
C7—C9—H9A	109.5	C22—C23—H23	117.4
C7—C9—H9B	109.5	C18—C23—H23	117.4
H9A—C9—H9B	109.5	C23—C24—C20	94.1 (4)
C7—C9—H9C	109.5	C23—C24—H24A	112.9
H9A—C9—H9C	109.5	C20—C24—H24A	112.9
H9B—C9—H9C	109.5	C23—C24—H24B	112.9
C1—C10—H10A	109.5	C20—C24—H24B	112.9
C1—C10—H10B	109.5	H24A—C24—H24B	110.3
